# Associations between intelligence, everyday executive functions, and symptoms of mental health problems in children and adolescents with mild intellectual disability

**DOI:** 10.1080/20473869.2023.2230412

**Published:** 2023-07-06

**Authors:** Sissel Gravråkmo, Lucy Henry, Alexander Olsen, Merete Glenne Øie, Stian Lydersen, Jo Magne Ingul

**Affiliations:** 1 Regional Centre for Habilitation at the Regional Centre for Child and Youth Mental Health and Child Welfare, Department of Mental Health, Faculty of Medicine and Health Sciences, Norwegian University of Science and Technology, Trondheim, Norway; 2 Department of Habilitation Services at the Children’s Clinic, St. Olav’s Hospital, Trondheim University Hospital, Trondheim, Norway; 3 Department of Language and Communication Science, City, University of London, UK; 4 Department of Psychology, Norwegian University of Science and Technology, Trondheim, Norway; 5 Department of Physical Medicine and Rehabilitation, St. Olav’s Hospital, Trondheim University Hospital, Trondheim, Norway; 6 Department of Psychology, University of Oslo, Oslo, Norway; 7 Regional Centre for Child and Youth Mental Health and Child Welfare, Department of Mental Health, Faculty of Medicine and Health Sciences, Norwegian University of Science and Technology, Trondheim, Norway

**Keywords:** mental health, executive function, intellectual disability, neurodevelopmental disorders, developmental disorders, symptoms of mental health problems

## Abstract

**Background:** Children and adolescents with intellectual disabilities have been found to have a higher prevalence of symptoms of mental health problems than the general population, but it is unclear whether these challenges are related to intelligence. The aim of this study was to explore the associations among symptoms of mental health problems, everyday executive functions and intelligence in this group.

**Methods:** Forty children and adolescents, pre-diagnosed with mild intellectual disability, were assessed for symptoms of mental health problems, intelligence, and everyday executive functions. The associations were explored using linear regression analyses.

**Results:** Symptoms of mental health problems were associated with everyday executive functions but not with intelligence. The prevalence of mental health problems within the group was three to four times higher than what is typically observed in the general population.

**Conclusion:** Although a remarkably high prevalence of symptoms of mental health problems was found among children and adolescents with pre-diagnosed mild intellectual disability, no relationship to intelligence was discovered in this population. Instead, a relationship between everyday executive functions and symptoms of mental health problems was found. Assessing everyday executive functions in children and adolescents with mild intellectual disability can provide valuable information about what support should be provided to prevent mental health problems in this population.

## Introduction

Studies have indicated that executive functions are associated with ‘[…] just about every aspect of life’, including mental health (Diamond 2013). In the general population, executive difficulties have been reported to be both a risk factor and a result of mental health problems among children ages 9–12 years (Romer and Pizzagalli 2021), suggesting a reciprocal relationship. However, it is not clear how this applies to children and adolescents with intellectual disability. For this reason, the current study investigated the relationship between symptoms of mental health problems and everyday executive functions in a group of children and adolescents with pre-diagnosed mild intellectual disability.

Executive functions refer to top-down cognitive processes that are required for the regulation of other cognitive processes, behaviour, and emotions (Diamond 2013). They are usually referred to as higher-order functions (Goldstein *et al*. 2014) and often include the process of goal formation, planning, and the ability to carry out plans in an effective manner (Jurado and Rosselli 2007). According to one prominent theoretical position, there is support for three main executive functions: shifting (cognitive flexibility), updating (working memory), and inhibition or impulse control (Miyake *et al*. 2000). Executive functions have traditionally been assessed using performance-based tests or through questionnaires, for example, the Behavior Rating Inventory of Executive Function (BRIEF), which measures everyday executive function (Chan *et al*. 2008, Roth *et al*. 2014). Assessing everyday executive functions using the Behavior Rating Inventory of Executive Function allows for high ecological validity (Gioia *et al*. 2000).

There is a growing interest in the role of executive functions in the everyday lives of people with intellectual disabilities (Fidler and Lanfranchi 2022). There is evidence that individuals with intellectual disability perform under the level expected according to their mental age on assessments of executive functions (Spaniol and Danielsson 2022). Furthermore, findings indicate that intelligence and everyday executive functions are related but separate constructs in children with intellectual disability (Gravråkmo *et al*. 2022). There is, however, little current research exploring how executive functions and intelligence may be related to symptoms of mental health problems in children and adolescents with intellectual disability. The suggested role of executive functioning as an adaptive process mediating between stress and cognitive demand can prove to be especially relevant in the development of mental health problems in this population (Kluwe-Schiavon *et al*. 2016). This suggests a need for studies exploring these associations and the role of intelligence and executive functions in the development of symptoms of mental health problems among children and adolescents with intellectual disability.

In the current study, we define symptoms of mental health problems as emotional and behavioural problems that are measured by the Child Behavior Checklist Total Problems Scale (Achenbach and Rescorla 2001). Understanding the associations between mental health, intelligence and everyday executive functions is important, because the prevalence of symptoms of mental health problems among children and adolescents diagnosed with neurogenic disorders associated with intellectual disability is found to be significantly higher than in the general population (Rutter *et al*. 1970, Glasson *et al*. 2020). A systematic review and meta-analysis calculated a pooled prevalence estimate of symptoms of mental health problems of 49% (CI 95%: 46–51) in a population of children and adolescents (ages 6–21) with intellectual disability (Buckley *et al*. 2020). By contrast, in the general population, 13–14% of children and adolescents have been identified as having mental health problems (Sawyer *et al*. 2001, Polanczyk *et al*. 2015). Despite this, findings suggest that children and adolescents with intellectual disability experience reduced access to mental health services (Trollor 2014, Soltau *et al*. 2015, Whittle *et al*. 2019).

Based on the high prevalence estimates of symptoms of mental health problems among children and adolescents with intellectualy disability, the current study addresses the question if there is an association between these symptoms and intelligence. There have been conflicting reports regarding the relationship between the level of intellectual disability and the prevalence of symptoms of mental health problems (Buckley *et al*. 2020). Some studies have revealed minor variations in mental health problems across levels of intellectual disability (Gillberg *et al*. 1986, Strømme and Diseth 2000, Marino *et al*. 2019). However, Einfeld and Tonge (1996) reported a trend towards lower relative risk for mental health problems in children and adolescents with profound intellectual disability compared to those with less severe levels of disability. Still other reports have indicated fewer symptoms of mental health problems among adults with mild and profound intellectual disability, in contrast to more symptoms in the moderate and severe groups (Koskentausta *et al*. 2007, Hove and Havik 2010). In their meta-analysis, Buckley *et al*. (2020) reported no differences in symptoms of mental health problems across three levels of intellectual disability (i.e. mild, moderate, and severe). Based on these diverging results and the high prevalence of mental health problems in children and adolescents with intellectual disability compared to the general population (Buckley *et al*. 2020) it is particularly important to study potential associations between the essential constructs relevant for the development of mental health problems in people with intellectual disability.

### Aims

The primary aim of the current study was to explore the relationships between symptoms of mental health problems, everyday executive functions, and intelligence in children and adolescents with pre-diagnosed mild intellectual disability, examining whether intelligence or executive functions could best predict symptoms of mental health problems. Based on previous research in the general population, we expected to find a positive association between everyday executive difficulties and symptoms of mental health problems. We also wanted to explore the associations between mental health and two BRIEF executive function indices, the Metacognition Index (initiate, working memory, plan/organise, monitor, and organisation of materials) and the Behavior Regulation Index (inhibit, shift and emotional control). The secondary aim of the present study was to give estimations of symptoms of mental health problems in children and adolescents with pre-diagnosed mild intellectual disability. Based on previous research, we expected to find a higher prevalence than in the general population.

## Methods

### Participants and procedure

**Figure 1.**
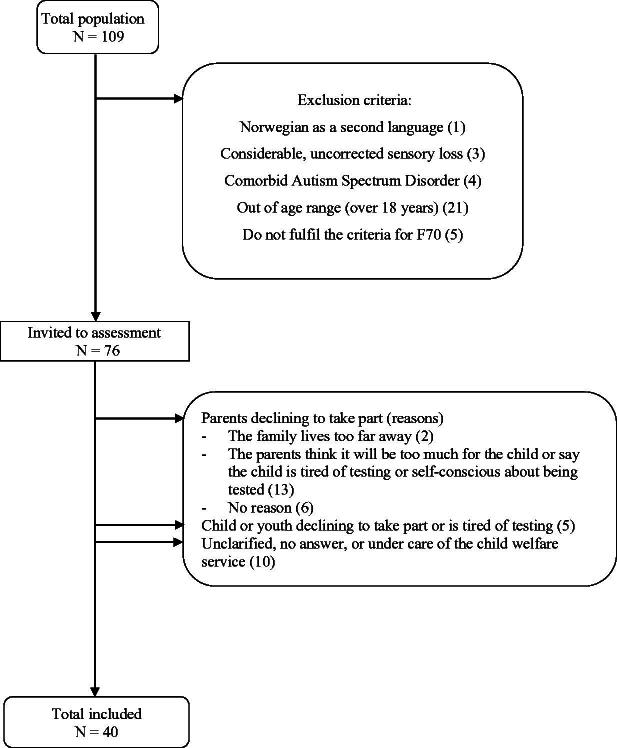
Flowchart illustrating subject recruitment and attrition. Adapted from “Associations between executive functions, intelligence and adaptive behaviour in children and adolescents with mild intellectual disability”, by S. Gravråkmo *et al*. 2022, Journal of Intellectual Disabilities, 1-13. © The Authors 2022 Adapted with permission DOI: 10.1177/17446295221095951. https://journals.sagepub.com/doi/10.1177/1744629522109595

A total of 76 children and adolescents pre-diagnosed with mild intellectual disability at the hospital habilitation clinics in the region of Central Norway were invited to the study. Exclusion criteria were: Having a co-existing diagnosis on the autism spectrum, not having Norwegian as a native language, and having large and uncorrected sensory loss; see Figure 1, adapted from Gravråkmo *et al*. (2022). The final sample included 40 children and adolescents (40% girls) in the age range of 10–17 years (mean 14.8; SD 2.1) with a pre-diagnosed mild intellectual disability and mixed aetiology; they participated in the study from 2018 to 2020. See Table 1 for characteristics of the participants and results on assessments.

**Table 1.** Descriptive statistics for the study participants. Number of available observations (*n*), mean (SD), and min–max or count (%).

Characteristics	*N*	mean (SD) or count (%)	min–max
Female sex	40	16.0 (40%)	
Age	40	14.8 (2.1)	10–17
Wechsler (FSIQ)	36	56.9 (11.1)	40–88
BRIEF (GEC)	37	68.5 (12.9)	40–90
BRIEF (BRI)	38	67.4 (14.7)	39–94
BRIEF (MI)	37	67.2 (12.0)	41–89
CBCL (TPS)	39	65.0 (9.0)	46–82

Wechsler: Wechsler Intelligence Scale for Children Fourth Edition (WISC-IV) and Wechsler Adult Intelligence Scale Fourth Edition (WAIS-IV), Full Scale IQ (FSIQ); BRIEF, Behavior Rating Inventory of Executive Function, Global Executive Composite (GEC), Behavior Regulation Index (BRI), Metacognition Index (MI); CBCL, Child Behavior Checklist from the Achenbach System of Empirically Based Assessment (ASEBA), Total Problems Scale (TPS) score.

Mild intellectual disability was already diagnosed by psychologists and medical doctors in specialised outpatient clinics based on the ICD-10 requirements and was a criterion for inclusion in the study (World Health Organization 2016). Following the ICD-10 criteria and the standardised procedures, the diagnosis requires that there be significant limitations (2 SD under the mean) on measures of intelligence and adaptive behaviours on valid assessment tools, such as the Wechsler Intelligence Scales and the Vineland Adaptive Behavior Scales, during the developmental period (World Health Organization 2016). Although it has been suggested that the ICD-10 has not been distinct enough regarding the requirement of co-occurring difficulties in adaptive functioning in the diagnosis of intellectual disability (Regionsenter for habiliteringstjenesten for barn og unge 2019), this distinction has been stated in the Norwegian national recommendations for child and adolescent psychiatry since 2010 (Gjærum 2016). Over the same time period, the DSM-5 has been explicit in the requirement of significant difficulties in adaptive behaviour and intelligence (American Psychiatric Association 2013).

The research followed the declaration of Helsinki and the study was approved by The Regional Committee for Medical Research Ethics – Southeast Norway REC 2009/932 and REC 2012/1976. Caregivers and participants gave informed assent and consent.

## Materials and measures

### Symptoms of mental health problems

Parents or caregivers, such as foster parents, completed the Child Behavior Checklist (CBCL) 6–18 years from the Achenbach System of Empirically Based Assessment (Achenbach and Rescorla 2001). The CBCL is a standardised report questionnaire used to assess symptoms of mental health issues, such as behavioural or emotional problems among children and adolescents. The CBCL is scored on eight statistically derived syndrome scales and three comprehensive scales: Internalising, Externalising, and Total Problems (Rescorla *et al*. 2012). The Internalising scale is composed of the Anxious/Depressed, Withdrawn/Depressed and Somatic Complaints syndrome scales meant to reflect a child’s tendency to internalise their emotional and behavioural problems and is used to assess a child’s emotional well-being. The Externalising scale is composed of the Aggressive Behaviour and Rule-Breaking Behaviour syndromes meant to represent conflicts with other people and with their expectations for children’s behaviour (Achenbach and Rescorla 2001).

For the current study, the Total Problems Scale (TPS) was used to measure overall symptoms of mental health problems including the following syndrome scales: emotional and behavioural problems related to anxiety disorders, mood disorders such as depression, somatic complaints, social problems, thought problems, attention problems, rule-breaking behaviours, and aggressive behaviours (Achenbach and Rescorla 2001). Results are given in *T*-scores with a mean of 50 and a standard deviation (SD) of 10, and higher scores on the CBCL indicate more problems. Internalising, Externalising, and Total Problems *T*-scores over 63 (above the 90^th^ percentile) represent symptoms of mental health problems of clinical significance, and scores in the range *T* = 60–63 (84^th^–90^th^ percentile) represent borderline symptoms (Achenbach and Rescorla 2001). *T*-scores below 60 are in the normal range.

Regarding its psychometric properties, the CBCL Total Problems Scale demonstrates good retest reliability (*r* = 0.94) and internal consistency (Cronbach’s alpha) of .97 (Achenbach and Rescorla 2001). Support is found for the content validity of this scale through many years of revisions and research; additionally, evidence indicates that the scale items can significantly discriminate between demographically matched referred and non-referred children (Achenbach and Rescorla 2001). The construct validity of the scale has been supported in many ways, including findings of significant associations with other assessment tools and with DSM criteria (Achenbach and Rescorla 2001).

Although the Norwegian population in general has been recognised as scoring relatively lower on the Total Problems Scale compared to other societies (Achenbach 2019), several studies have supported the use of the CBCL as a screening instrument in a Norwegian population (Nøvik 1999b, Ivanova *et al*. 2007, Jozefiak *et al*. 2012, Kornør and Jozefiak 2012). The construct validity of the Norwegian CBCL 4–18 is good (*N* = 949; RMSEA = .039) (Nøvik 1999a), supporting the concept validity of the Norwegian CBCL (Ivanova *et al*. 2007). The criterion validity has indicated promising sensitivity (40%–83%) and specificity (70%–94%), and the internal consistency of the Total Problems is also good, as measured by Cronbach’s alpha of *α* > .80 (Nøvik 1999b). A study using the CBCL 6–18 reported satisfactory internal consistency (Jozefiak *et al*. 2012). The correlation for the 2001 CBCL Total Problems Scale 6–18 and the 1991 CBCL Total Problems Scale 4–18 has been reported to be 1.0 (Achenbach and Rescorla 2001).

In the current study, the prevalence of symptoms of mental health problems was based on the percentage of participants scoring over the clinically significant cut-off on the CBCL Total Problems Scale (TPS), and in the regression analyses, the TPS score was used as a continuous variable, as recommended by Lydersen (2015). Evaluations have concluded that the CBCL is suited for use in the population of children and adolescents (6–13 years) with mild intellectual disability (Koskentausta *et al*. 2004) as well as for school-age children with Down syndrome (Esbensen *et al*. 2018).

### Everyday executive function

The Behavior Rating Inventory of Executive Function (Gioia *et al*. 2000, Fallmyr and Egeland 2011), a parent-report questionnaire for children and adolescents in the age range of 5–18 years, was completed by parents or caregivers (*n* = 37). For the parent-completed form, the original psychometric reports of the Global Executive Composite, the overall summary measure of everyday execution functions, indicated a high internal consistency of *α* = .98 and *α* = .97, for the clinical and normative samples, respectively. Retest-coefficients ranged from .76 to .88 and from .72 to .84 for the normative sample and clinical sample, respectively (Gioia *et al*. 2000). Most of the items in the BRIEF have been demonstrated to have high interrater agreement, indicating that the items within each scale adequately express the intended executive function domain, supporting the content validity of the BRIEF (Gioia *et al*. 2000). The parent form of the BRIEF has demonstrated correlations to other behavioural measures, with the exception of emotional functioning, providing evidence for convergent and divergent validity (Gioia *et al*. 2000). Children with mild to moderate intellectual disability were found to be rated similarly to matched controls on the Global Executive Composite (Gioia *et al*. 2000). The Norwegian form using American norms has been found to be valid, with good psychometric properties for the general population (Sørensen and Hysing 2014). The BRIEF parent-report questionnaire has also been found to have good psychometric properties and be suited for use for children and adolescents (ages 6–18) with Down syndrome (Esbensen *et al*. 2019).

The Global Executive Composite (GEC) was used in the current study, as it is a summary measure of the clinical scales of everyday execution functions – inhibit, shift, emotional control, initiate, working memory, plan/organise, organisation of materials, and monitor. Scores on the Behavior Regulation Index (BRI) and Metacognition Index (MI) were also used as measures of everyday executive functions. The Behavior Regulation Index is related to the child’s ability to regulate emotions and behaviour as well as the capacity to shift attention. The Metacognition Index includes initiating, organising, and monitoring actions as well as working memory (Gioia *et al*. 2000). Higher scores on the BRIEF scales indicate more significant problems in everyday executive function behaviour, and a *T*-score of 65 (corresponding to 1.5 SDs above the mean of 50) or more indicates problems of clinical significance (Gioia *et al*. 2000).

### Intelligence

For the majority of participants, assessments of intelligence had been carried out more than two years previously. Therefore, for these participants new tests were administered (*n* = 38), using the Wechsler Intelligence Scale for Children, Fourth Edition (WISC-IV, *n* = 36; Wechsler 2009), or the Wechsler Adult Intelligence Scale, Fourth Edition (WAIS-IV, *n* = 2; Wechsler 2011). For the participants with tests results less than two years old (*n* = 2), existing results were used. As a measure of intelligence, the Full-Scale Intelligence Quotient (FSIQ) from the WISC-IV and WAIS-IV was used with IQ-standard values (mean (M) = 100 and standard deviation (SD)=15. Regarding psychometric properties of the Norwegian version, the WISC-IV has been found to replicate the American and Swedish versions with an across-age average reliability coefficient (Fisher’s *z* transformation) of *r* = 0.97 for the full-scale IQ (FSIQ) (Wechsler 2003, Wechsler 2009), which also holds for the Scandinavian WAIS-IV (*r* > 0.95: Wechsler 2011).

Considering that mild intellectual disability was an inclusion criterion in this study, the Wechsler FSIQ repeat assessments revealed wider variability than expected (IQ scores between 40–88). Inclusion according to the original evaluations was, however, retained, acknowledging the comprehensive process of diagnosing intellectual disability, not exclusively relying on measures of intelligence (World Health Organization 2016). To provide uniformity in the administration and interpretation, the repeat assessments were used in the analyses.

### Statistical analyses

Linear regression analyses were conducted with the CBCL (TPS) as the dependent variable, and the Wechsler (FSIQ) and the BRIEF (GEC) as predictors, one at a time and simultaneously. Linear regression analyses were also conducted with the CBCL (TPS) as the dependent variable and the Behavior Regulation Index (BRI) and the Metacognition Index (MI) from the BRIEF as predictors, one at a time. The analyses were conducted both unadjusted and adjusting for sex and age, since it is possible that there is an effect of age and sex in the population we study. The measured T-scores and IQ were normalised according to age and/or sex in a general population. Missing values were handled using available case analysis, which means that, in every analysis, all cases with data on the relevant variables were included. Normality of residuals was confirmed by visual inspection of QQ-plots. Two-sided *p*-values < 0.05 were specified to indicate statistical significance, and 95% confidence intervals are reported when relevant.

## Results

### Descriptive statistics

Table 1 provides descriptive statistics for all participants. In the sample (*n* = 40), a total of 21 participants (52.5%) had a CBCL Total Problems Scale (TPS) score in the clinical range, and six (15%) had scores in the borderline range, meaning that a combined 27 (67.5%) exhibited evidence for symptoms of mental health problems. For internalising problems, 15 (37.5%) had scores in the clinical range and five (12.5%) in the borderline range, meaning that a total of 20 (50%) had noteworthy difficulties. For externalising problems, 14 (35%) had scores in the clinical range and two (5%) in the borderline range for a total of 16 (40%) participants with noteworthy difficulties in this area.

On the Global Executive Composite (GEC) of the BRIEF, in the current sample, 22 participants (55%) had a score in the clinical range; for the Behavior Regulation Index (BRI) of the BRIEF 24 (60%) had a score in the clinically significant range; and for the Metacognitive Index (MI) of the BRIEF, 21 (52.5%) had a score in the clinically significant range.

### Regression analyses

Results of linear regression analyses are presented in Tables 2 and 3. The Wechsler Full-Scale Intelligence Quotient (FSIQ) was not a significant predictor for the CBCL Total Problems Scale (TPS) score (regression coefficient of −0.01, *p* = .96), whereas the BRIEF Global Executive Composite (GEC) was a significant predictor (regression coefficient of 0.59, *p* < .001). As Table 2 reveals, the regression coefficients were similar in the analyses with one predictor at a time and with both predictors included in the model at the same time. In the combined model, the BRIEF Global Executive Composite (GEC) and the Wechsler Full-Scale IQ (FSIQ) accounted for 65% of the variance of the CBCL Total Problems Scale (TPS) score with a significant *p*-value of < .001 (adjusted R square = .65). Analyses adjusted for age and sex provided substantially the same results; see Supplementary table.

**Table 2.** Regression analyses with CBCL Total problems Scale (TPS) score as dependent variable and Wechsler Full-Scale IQ (FSIQ) and BRIEF Global executive Composite (GEC) as predictors.

Predictors	*n*	regression coefficient*	95% CI	*p*
One at a time				
Wechsler (FSIQ)	36	−0.007	−0.29 to 0.27	.96
BRIEF (GEC)	37	0.59	0.45 to 0.73	< .001
Simultaneously				
Wechsler (FSIQ) and BRIEF (GEC)	35			
Wechsler (FSIQ)		0.07	−0.10 to 0.23	.43
BRIEF (GEC)		0.58	0.43 to 0.72	< .001

Wechsler: Wechsler Intelligence Scale for Children, Fourth Edition (WISC-IV) and Wechsler Adult Intelligence Scale, Fourth Edition (WAIS-IV), Full-Scale IQ (FSIQ); Behavior Rating Inventory of Executive Function (BRIEF), Global Executive Composite (GEC); Child Behavior Checklist (CBCL) from the Achenbach System of Empirically Based Assessment (ASEBA), Total Problems Scale (TPS) score.

*Unstandardised regression coefficient.

**Table 3.** Regression analyses with the CBCL Total problems Scale (TPS) score as the dependent variable and BRIEF indices (BRI and MI) as predictors.

Predictors	*n*	Regression coefficient*	95% CI	*p*
BRIEF (BRI)	38	0.36	0.21 to 0.52	< .001
BRIEF (MI)	37	0.25	0.058 to 0.45	.013

Behavior Rating Inventory of Executive Function (BRIEF), Behavior Regulation Index (BRI), Metacognition Index (MI); Child Behavior Checklist (CBCL) from the Achenbach System of Empirically Based Assessment (ASEBA), Total Problems Scale (TPS) score.

*Unstandardised regression coefficient.

To explore whether the Metacognition Index (MI) or the Behavior Regulation Index (BRI) assessed by the BRIEF, or both, could predict a CBCL Total Problems scale (TPS) score, a regression analysis with these as independent variables was conducted. Results are presented in Table 3. Both regression coefficients were statistically significant, and the sum of the regression coefficients was approximately equal to the coefficient for the BRIEF (GEC) in Table 2. Therefore, we can conclude that both indices contributed substantially to predict symptoms of mental health issues in this group.

## Discussion

In this study, as predicted, we observed a significant association between symptoms of mental health problems and difficulties with everyday executive functions in children and adolescents pre-diagnosed with mild intellectual disability. This is in line with previous findings in the general population of children and adolescents (Romer and Pizzagalli 2021). We found that the Metacognition Index (MI) and the Behavior Regulation Index (BRI) from the BRIEF both contributed to this association. Neither of the indices seemed to be more closely related to symptoms of mental health issues than the other. Furthermore, no relationship was observed between symptoms of mental health problems and intelligence in this group of children and adolescents. This may suggest that, despite more evidence of mental health problems in children and adolescents with intellectual disabilities, their mental health may also be significantly related to higher-order functions necessary for human regulation and goal-directed behavior (Miyake *et al*. 2000, Goldstein *et al*. 2014).

Considering that a reciprocal causal relationship has been reported between executive difficulties and mental health problems in the general population (Romer and Pizzagalli 2021), there is a need for more knowledge about how this applies specifically in populations with intellectual disabilities. Evidence from research in populations with identified syndromes, such as Down and Williams syndromes, suggests that differences in mental health problems might be related to syndrome-specific genetics and characteristic profiles in executive functioning (Carney *et al*. 2013, Glasson *et al*. 2020). For children and adolescents with mild intellectual disability, the most prevalent of the intellectual disabilities, the aetiology is often unknown (Patel *et al*. 2020). This means that the assessment of executive functions in everyday life could provide additional clinically and educationally meaningful information that may lead to a deeper understanding of the development and treatment of the individual’s mental health.

Given the results from the current study, it could be useful to look to the stress-vulnerability hypothesis, whereby executive functioning is seen as key to the regulation between activation, or stress, and cognitive demand (Kluwe-Schiavon *et al*. 2016). Although necessary for activation in encountering new challenges (Kluwe-Schiavon *et al*. 2016), stress can become overpowering when there is imbalance in this dynamic, and individuals with intellectual disability might be especially vulnerable during this process. Over time, difficulties mediating between activation and cognitive demand could interact with, or even influence, symptoms of mental health problems. This model could offer a perspective on how executive functions could be of significance for the development of symptoms of mental health problems in children and adolescents with intellectual disability. This is a hypothesis that could be explored in further research, also corresponding with studies in the general child and adult population that have demonstrated cognitive challenges, including executive function difficulties, across a wide range of mental health disorders (Abramovitch *et al*. 2021). Additionally, it has been suggested that training cognitive functions such as rule-use and efficient reflection could potentially help children at risk (Zelazo 2015).

As for the secondary aim, the current results indicated a considerable prevalence of symptoms of mental health problems (53%) in this group of children and adolescents with intellectual disability. This corresponds with earlier findings reporting a prevalence rate of 49% for mental health problems in populations of children and adolescents with intellectual disability (Buckley *et al*. 2020). This is a significantly elevated prevalence compared to that reported for children and adolescents in the general population (14%, 95% CI = [13, 15]) (Sawyer *et al*. 2001, Polanczyk *et al*. 2015). The high prevalence estimates of mental health problems in populations with intellectual disabilities underline the importance of correctly and specifically recognising, assessing, and treating these problems (Matson and Shoemaker 2011).

Mental health problems are often not identified and, therefore, are left untreated in those with intellectual disabilities (Einfeld and Tonge 1996, Trollor 2014, Soltau *et al*. 2015, Whittle *et al*. 2019, Buckley *et al*. 2020), possibly because of ‘atypical presentation, communication difficulties, lack of continuity of care, no valid diagnostic system, difficulties in accessing care, and inadequate training of health professionals’, as described by Krahn *et al*. (2006). Problems related to organisation, lack of services, and the quality of services have been discussed as potential causes for the lack of professional help for individuals with intellectual disability (Whittle *et al*. 2018). Further, it has been demonstrated that the main problem is not difficulty related to the recognition of symptoms of mental health problems by individuals with intellectual disability themselves or by caregivers; rather, it is symptoms being misinterpreted as behavioural issues, in other words, the phenomenon of ‘diagnostic overshadowing’ when in contact with specialised care (Whittle *et al*. 2019). Diagnostic overshadowing means that mental health problems can be misinterpreted or ignored in individuals with intellectual disabilities (Jopp and Keys 2001, Mason and Scior 2004, Rush *et al*. 2004, Krahn *et al*. 2006). Additionally, delays in access to treatment and debates about where youth with intellectual disabilities should acquire help have been reported by Walton *et al*. (2022). Having mental health problems that are not recognised and left untreated can lead to unnecessary suffering and cost for the individuals themselves, their families, and society at large (Polder *et al*. 2002).

An important step towards helping children and adolescents with intellectual disability is to ensure that mental health symptoms are not overlooked or misinterpreted as part of the diagnosis of intellectual disability (Spengler *et al*. 1990, White *et al*. 1995, Jopp and Keys 2001, Rush *et al*. 2004, Manohar *et al*. 2016). It is also vital that children and adolescents with intellectual disabilities are referred to services that offer assessment and treatment for mental health problems (Mason 2007, Nylander *et al*. 2016, Pelleboer-Gunnink *et al*. 2019). It is important to work towards easy access to mental health services that have competency regarding both intellectual disability and mental health. In this regard, refusals by clinicians, based on feelings of incompetency should be avoided (Soltau *et al*. 2015), ensuring that professionals in mental health care know that symptoms of mental health problems in children and adolescents with mild and moderate intellectual disability are similar to those in the general population (Gillberg *et al*. 1986, Matson and Shoemaker 2011). There is a need for further research to address questions related to the relationship between executive functions and symptoms of mental health problems in children and adolescents with intellectual disability, as well as questions regarding the prevalence of and access to assessment and treatment for mental health problems in this population.

### Strengths and limitations

Using a parent-rating questionnaire such as the CBCL can be considered to be a strength in this study, given that symptoms of mental health problems in children and adolescents with mild intellectual disability have been demonstrated to be analogous to those in the general population (Matson and Shoemaker 2011). Furthermore, the CBCL has been recognised as suited to assess symptoms of mental health problems in children and adolescents with intellectual disability (Koskentausta *et al*. 2004, Esbensen *et al*. 2018). It has also been suggested that mental health symptoms can be better detected by symptom phenotypes than by formal psychiatric diagnosis, which Buckley *et al*. (2020) describe as possibly not capturing the range of mental health problems in children and adolescents with intellectual disability. Another strength is the use of a parent-report measure of executive functions, such as the BRIEF, which has been demonstrated to be appropriate for use for people with intellectual disability while also retaining its psychometric properties (Fidler and Lanfranchi 2022). The BRIEF, as a parent-rated questionnaire, captures executive functions in real-life settings providing good ecological validity (Silver 2014).

A possible limitation of this study is that intelligence was measured using performance-based measures, while everyday executive functions and symptoms of mental health problems were assessed using parent-rated questionnaires. Furthermore, two parent-rated questionnaires can be more likely to be related to each other because of an inherent “bias in style” on the part of the parent. Nevertheless, using standardised assessment methods that are frequently used in clinical settings is a strength in this study. There is always a possibility that using measures like the CBCL and the BRIEF, that are not specifically adapted for this population, could lead to higher problem scores related to the divergence between the actual age and the developmental age in this population with a diagnosis of intellectual disability. Another limitation is the relatively small number of participants, which sets restrictions regarding the statistical power of the study and, as a result, influences the possibilities for exploring additional indices and more fine-grained subscales of the measures, including more individualised profiles. A final possible limitation is related to the aim of investigating mental health in youth with mild intellectual disability. This limits the variance (FSIQ: 40-88), and hence reduces the possibility to obtain statistically significant correlations between mental health and intelligence in this sample.

## Conclusions

The main finding in the current study was a significant relationship between symptoms of mental health problems and everyday executive functions in a group of children and adolescents with pre-diagnosed mild intellectual disability, a relationship that was not evident with intelligence. Furthermore, results confirmed previous findings of three to four times higher prevalence estimates for symptoms of mental health problems among children and adolescents with intellectual disability, compared to the general population. If further research confirms a reciprocal relationship between everyday executive functions and symptoms of mental health problems, a practical implication could be that providing support for executive functions in daily life may offer a way to support the mental health of children and adolescents with intellectual disability.

## Supplementary Material

Supplemental Material

## Data Availability

Due to ethical restrictions, supporting data is not available.
