# Supplementary material for: Associations between intelligence, everyday executive functions, and symptoms of mental health problems in children and adolescents with mild intellectual disability
Source: Int J Dev Disabil. 2023 Jul 6;71(2):256–65. doi: 10.1080/20473869.2023.2230412 (PMC11843635; doi:10.1080/20473869.2023.2230412)
Supplement: Supplemental Material [file YJDD_A_2230412_SM6635.docx]

# **Supplementary table**

# TABLE 4 | Supplementary table. Regression analyses with the CBCL Total Problems Scale (TPS) score as the dependent variable and Wechsler Full-Scale IQ (FSIQ), BRIEF Global Executive Composite (GEC), sex, and age as predictors.

| Predictors | *n* | *regression coefficient** | *95%* CI | *p* |
| --- | --- | --- | --- | --- |
| *Simultaneously* |  |  |  |  |
| Wechsler (FSIQ) and  BRIEF (GEC) | 35 |  |  |  |
| Wechsler (FSIQ) |  | 0.085 | -0.10 to 0.27 | .35 |
| BRIEF (GEC) |  | 0.57 | 0.42 to 0.72 | < .001 |
| Sex |  | 1.41 | -2.75 to 5.57 | .49 |
| Age |  | -0.12 | -1.04 to 0.80 | .79 |

Wechsler: Wechsler Intelligence Scale for Children, Fourth Edition (WISC-IV) and Wechsler Adult Intelligence Scale, Fourth Edition (WAIS-IV), Full-Scale IQ (FSIQ); Behavior Rating Inventory of Executive Function (BRIEF), Global Executive Composite (GEC); Child Behavior Checklist (CBCL) from the Achenbach System of Empirically Based Assessment (ASEBA), Total Problems Scale (TPS) Score

*Unstandardised regression coefficient
